# *Carex jianfengensis* (*Carex* sect. *Rhomboidales*, Cyperaceae), a New Species from Hainan, China

**DOI:** 10.1371/journal.pone.0136373

**Published:** 2015-09-23

**Authors:** Hubiao Yang, Xiaoxia Li, Wenqiang Wang, Changjun Bai, Guodao Liu

**Affiliations:** 1 Tropical Crops Genetic Resources Institute, Chinese Academy of Tropic Agricultural Sciences, Danzhou, Hainan, People's Republic of China; 2 Environment and Plant Protection Institute, Chinese Academy of Tropical Agricultural Sciences, Haikou, Hainan, People's Republic of China; National Institute for Viral Disease Control and Prevention, CDC, CHINA

## Abstract

A new species of *Carex* sect. *Rhomboidales*, *C*. *jianfengensis*, is described and illustrated from Hainan, China. The new species is similar to *C*. *zunyiensis* but differs in having involucral bracts sparsely hispid and with ca.1 cm long sheaths; inflorescence with 4 spikes, terminal spike ca. 2.5 cm long, lateral spikes 2–3.5 × 0.7–1 cm; staminate glumes narrowly ovate, ca. 5 mm; pistillate glumes triangular-lanceolate, 5–7 mm; perigynia 6–8 × 3 mm and pubescent on veins; nutlet 4–5 mm long, rhombic-ovoid, trigonous, base with shortly stipitate, apex abruptly contracted into a erect short beak, and not expanding into an annulate orifice.

## Introduction

The genus *Carex* L. is one of the largest genera of vascular plants, comprising about 2,000 species distributed almost worldwide in various habitats[1–5]. 527 species in three subgenera and 69 sections were recorded in recently published Flora of China. Recently, additional 18 species have been reported[6–18].

The genus *Carex* is clearly distinguished from other genera of the Cyperaceae in having consistently unisexual flowers and a perigynium, the latter a sac-like structure of prophyllar origin that surrounds the naked gynoecium[19]. *Carex* has been divided into subgenera in a number of ways based on the following characters: stigma number, inflorescence structure and distribution of staminate and pistillate flowers within the spikes. The most influential classification was that of Georg Kükenthal who recognized four subgenera: *Carex* subg. *Carex*, *C*. subg. *Indocarex*, *C*. subg. *Vignea* and *C*. subg. *Primocarex*. Subsequently, *C*. subg. *Indocarex* and *C*. subg. *Primocarex* were reclassified as *C*. subg. *Vigneastra* [20]. This classification was widely followed by most authors[2,21–25].

*Carex* sect. *Rhomboidales* belongs to *C*. subg. *Carex* and is characterized by long-sheathing bracts with short blades, trigonous, rhombic to ovoid perigynia with columniform bidentate beaks at the apex, and obovoid or ovoid, trigonous nutlets that are constricted in the middle part and mitrate or hastate at the apex[5,20]. The section consists of 41 species, mainly distributed in eastern Asia, with 36 species being native to China and 4 species in Hainan Island. [2, 7–9,11–14,16, 21,26–28,]. Hainan Island is located at the southern part of China, at the northern edge of tropical Asia, with about 4 100 vascular plant species. To date, 27 species of *Carex* have been reported from Hainan[6,14–16,29,].

During an investigation of the flora of Jianfeng Ling Nature Reserve in 2014, a novel species of *Carex* was collected and is here recognized as a new species in sect. *Rhomboidales*. Morphologically, the new species has affinities with *Carex zunyiensis* Tang & F.T. Wang.

## Materials and Methods

### Ethics statement

The new species reported in this work is collected from Jianfeng Ling Nature Reserve which is protected by the Forestry Bureau of Hainan. Permissions to visit location and field activities were obtained from Jianfeng Ling Nature Reserve Administration Bureau.

### Morphological observations

The morphological description is based on examination of fresh and dried specimens. Details of the staminate terminal spike, the pistillate lateral spikes, the pistillate glume, the perigynium and the nutlet were examined and photographed under a stereomicroscope (Olympus SZX16-6156). The shapes of perigynium and nutlet were observed using a Philips XL-30E scanning electron microscope. The studied specimens (one holotype, four isotypes, two paratypes) were deposited in the herbaria of the South China Botanical Garden, the Chinese Academy of Sciences (IBSC), and the Tropical Crops Genetic Resources Institute, Chinese Academy of Tropical Agricultural Sciences (TCGRI; not listed in Thiers 2008[30]).

### Nomenclatural Acts

The electronic version of this article in Portable Document Format (PDF) in a work with an ISSN or ISBN will represent a published work according to the International Code of Nomenclature for algae, fungi, and plants, and hence the new names contained in the electronic publication of a PLOS ONE article are effectively published under that Code from the electronic edition alone, so there is no longer any need to provide printed copies.

In addition, new names contained in this work have been submitted to IPNI, from where they will be made available to the Global Names Index. The IPNI LSIDs can be resolved and the associated information viewed through any standard web browser by appending the LSID contained in this publication to the prefix http://ipni.org/. The online version of this work is archived and available from the following digital repositories: PubMed Central, LOCKSS.

## Results

**Fig 1:**
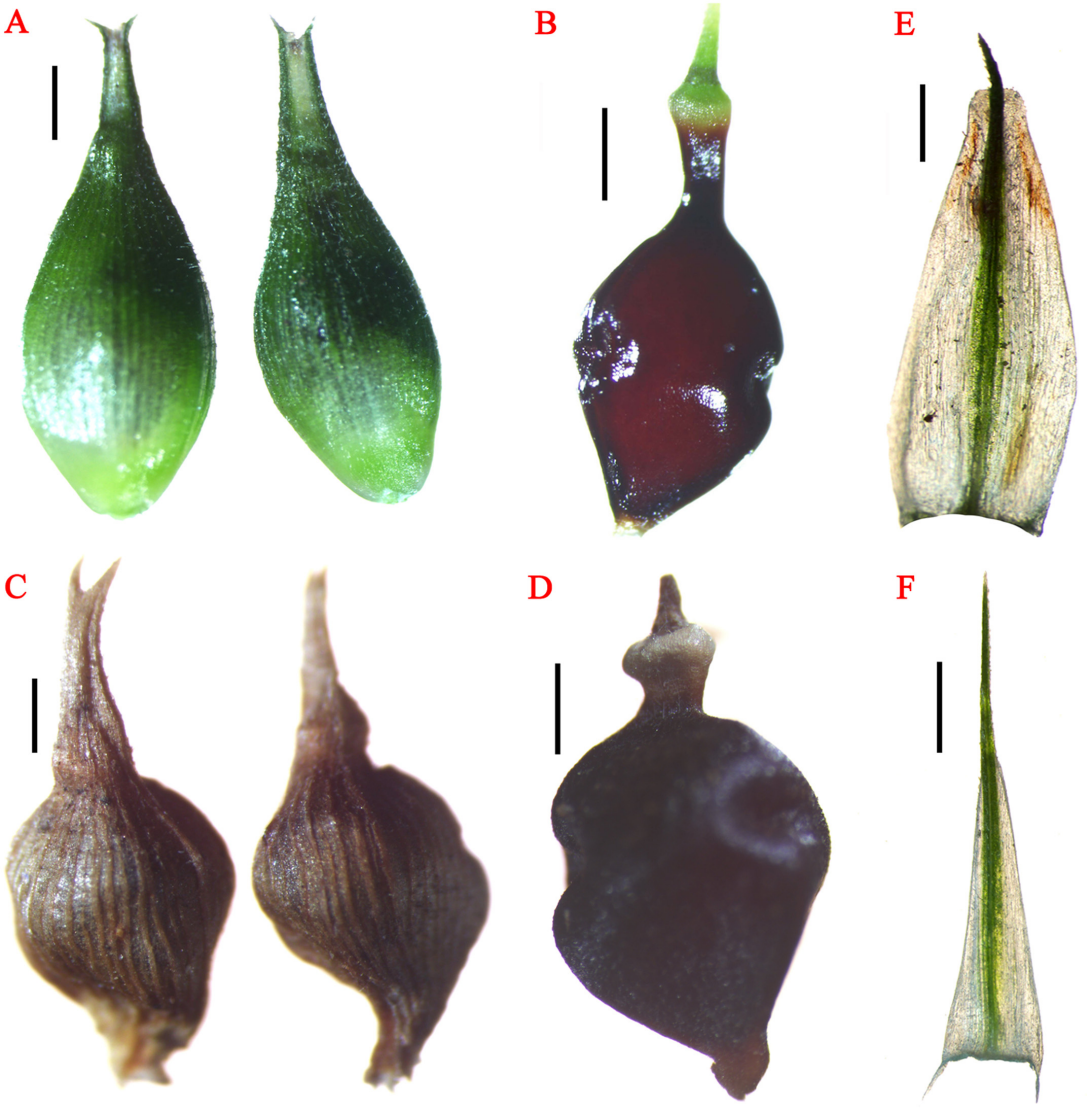
*Carex jianfengensis* A. Perigynia, B. Nutlet, E. Staminate glume, F. Pistillate glume; *C*. *zunyiensis* C. Perigynia. D. Nutlet. (*C*. *zunyiensis* from IBSC). Scale bars = 1 mm.

**Fig 2:**
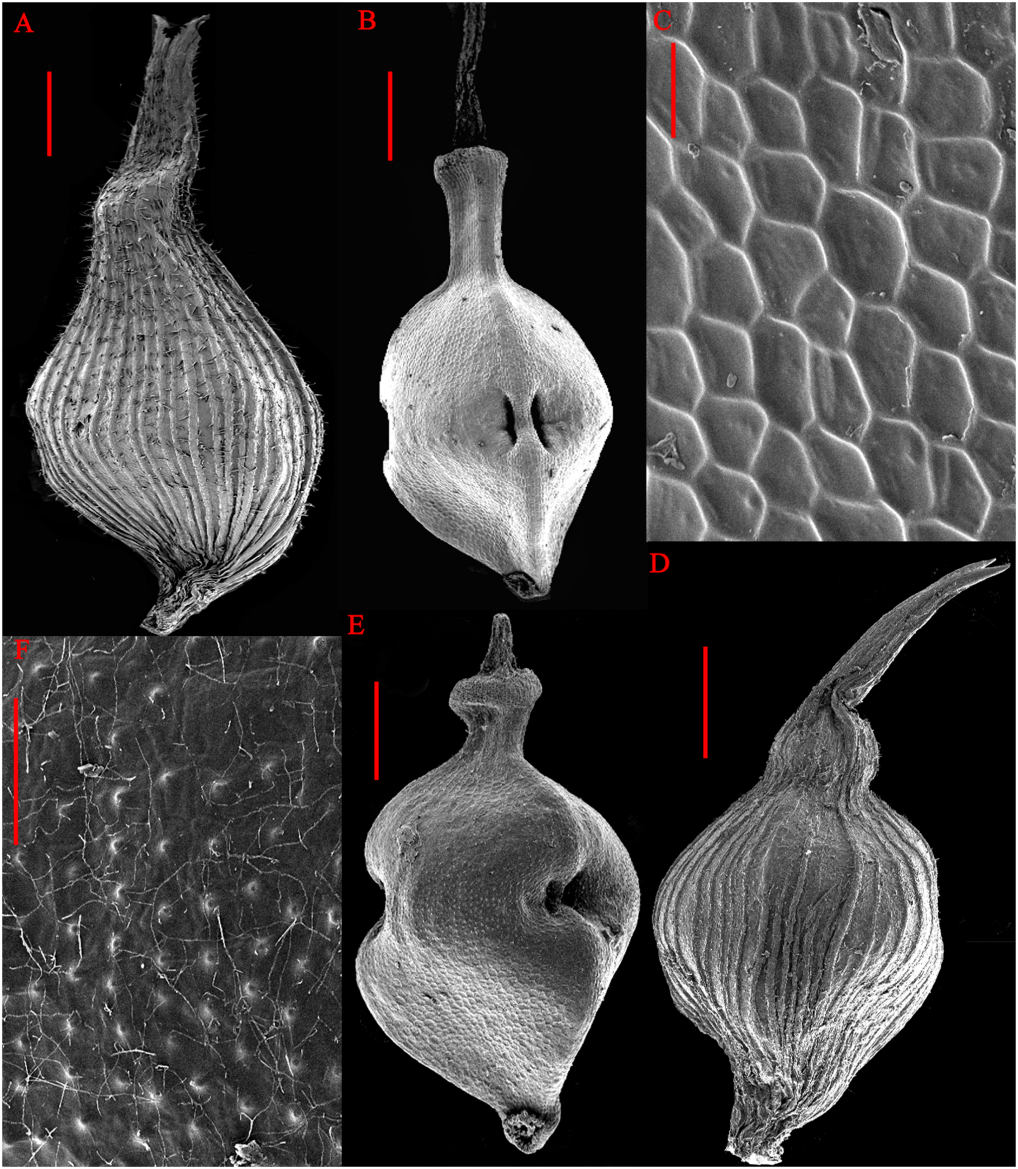
*Carex jianfengensis* A. Perigynium, B. Nutlet, C. Surface sculpturing; *C*. *zunyiensis* D. Perigynium, E. Nutlet, F. Surface sculpturing. (*C*. *zunyiensis* from IBSC). Scale bars: A. B. D. E = 1 mm; C = 50 um; F = 100 um.

The new species is most similar to *C*. *zunyiensis* based on the shape of the leaves, the very short culms and the subbasal and approximate spikes, but differs sufficiently to be recognized as a new species in morphological features (Table 1, Figs 1 and 2).

**Table 1:** Morphological comparison between *Carex jianfengensis* and *C*. *zunyiensis*. (Figs 1 and 2).

Character	*C*. *jianfengensis*	*C*. *zunyiensis*
**Sheaths**	2−7 cm long and with purple vertical stripes	basal ones dark brown disintegrating into fibers, upper ones slightly scabrous on the margin
**Bracts**	sparsely hispid and with ca. 1 cm long sheaths	glabrous, sheathless
**Inflorescence**	spikes 4 and occasionally 1 lateral spike arising from the culm base	spikes 4−7
**Spikes**	terminal spike ca. 2.5 cm long; lateral spikes 2−3.5 cm long	terminal spike 3.5−5 cm long; lateral spikes 3−5 cm long
**Glumes**	staminate glumes narrowly ovate; pistillate glumes triangular-lanceolate, 5−7 mm long, green 1-veined, costa excurrent into a short awn ca. 2 mm long	staminate glumes lanceolate; pistillate glumes lanceolate, ca. 4 mm long, 3-veined, costa excurrent into a scabrous mucro at sharp apex
**Perigynia**	6−8 mm; densely hispid	4−5 mm; sparsely hispid
**Nutlets**	rhombic-ovoid, with angles constricted at the middle, base shortly stipitate, apex abruptly contracted into an erect short beak and not expanding into an annulate orifice; epidermal cells with irregularly 4-5-gonal, central no silica body	ovate-elliptic with angles constricted above, apical beak short, curved, expanding into an annulate orifice; epidermal cells with irregularly 5-6-gonal, central with 1 silica body

## Taxonomic Treatment

### *Carex jianfengensis* H.B. Yang, X.X. Li & G.D. Liu sp. nov. (Figs 1–4)

**Fig 3:**
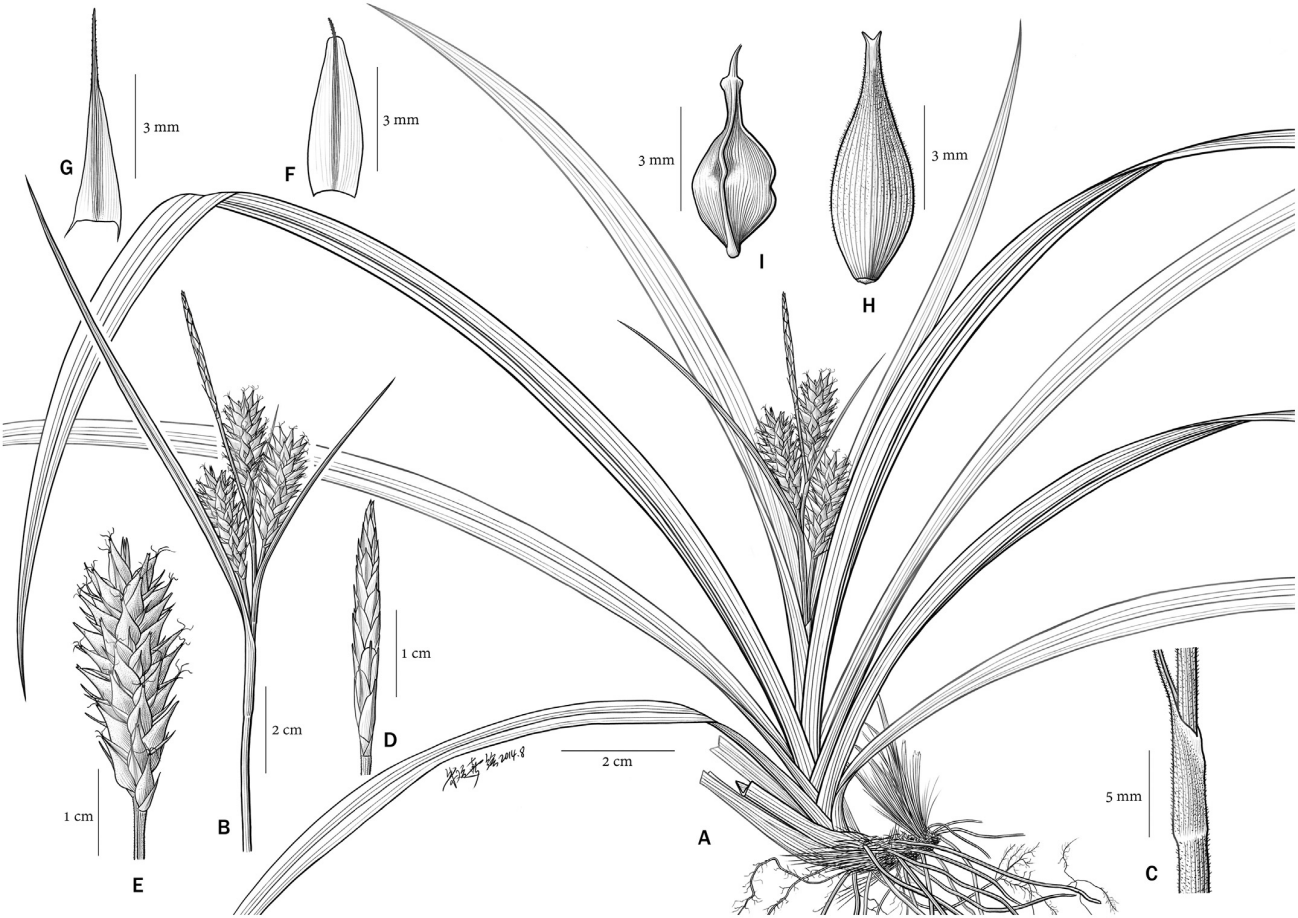
*Carex jianfengensis* A. Habit; B. Inflorescence; C. Bract sheaths; D. Terminal staminate spike; E. Lateral pistillate spike; F. Staminate glume; G. Pistillate glume; H. Perigynium; I. Nutlet.

**Fig 4:**
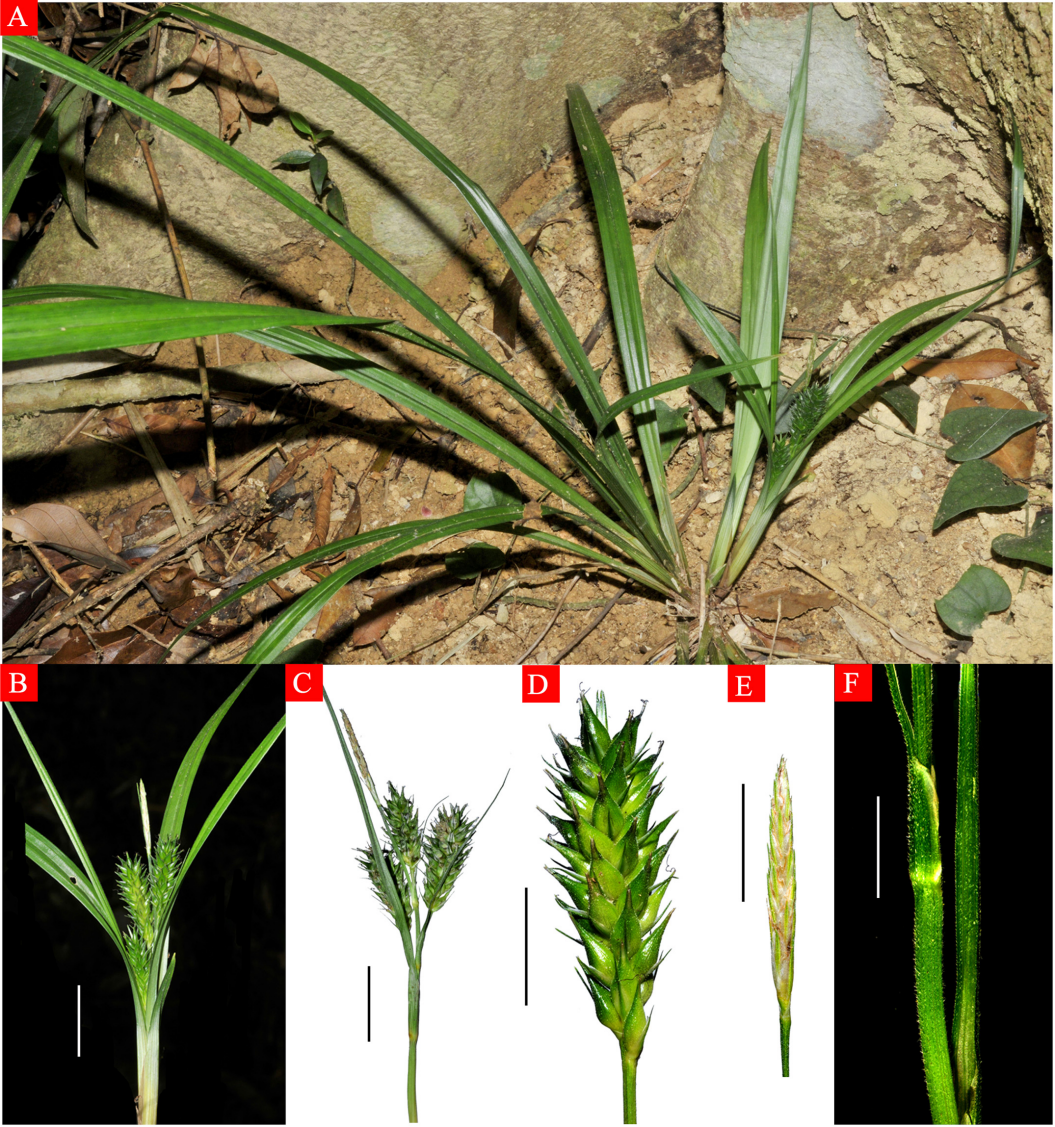
*Carex jianfengensis* A. Habit, B. Inflorescence, C. Inflorescence, D. Lateral pistillate spike, E. Terminal staminate spike, F. Bract sheaths.

The new species is similar to *C*. *zunyiensis*, but differs by having sparsely hispid involucral bracts and sheaths, inflorescence with 4 spikes and occasionally 1 lateral spike arising from the culm base,terminal spike ca. 2.5 cm long, lateral spikes 2–3.5 × 0.7–1 cm, staminate glumes narrowly ovate, ca. 5 mm long, truncate at apex, green 3-veined costa excurrent into a short awn ca. 0.6–0.8 mm long, pistillate glumes triangular-lanceolate, 5–7 mm long, green 3-veined costa excurrent into a short awn ca. 2 mm long, perigynium 6–8 × 3 mm, nutlet rhombic-ovoid, base shortly stipitate, short beak erect and not expanding into an annulate orifice.

#### Type

—CHINA. Hainan: Ledong County, Jianfeng Ling Nature Reserve, under forest, alt. 700−900 m, 17 March 2014, *Yang Hubiao 20140317001* (holotype, IBSC; isotype, IBSC; TCGRI three duplicates).

Perennial; rhizome short, covered with fibrous remains of old leaf sheath. Culms central, 2−4 × ca. 0.2 cm, significantly shorter than leaves, hiding in leaf sheaths, trigonous. Leaves basal, sheathed; blades linear, 25−65 × 1−1.6 cm, veins 3-ranked, green, flat, glabrous on both surfaces, apex acuminate; sheaths 2−7 cm long, with purple vertical stripes, basal ones bladeless. Involucral bracts slightly surpassing inflorescence, leaf-like, sparsely hispid, sheathed; sheaths ca. 1 cm long, sparsely hispid. Inflorescence of spikes racemose, with 4 spikes, approximate, occasionally 1 lateral spike arising from the culm base. Terminal spike staminate, trigonous-cylindrical, ca. 2.5 cm long; peduncle 2−3.5 cm long, sparsely hispid. Lateral spikes pistillate, cylindrical, 2−3.5 × 0.7−1 cm, densely flowered, with peduncle 1−3 cm long; occasionally peduncle up to 7 cm long when lowest lateral spike arises from the culm base. Staminate glumes membranous, glabrous, narrowly ovate, ca. 5 mm long, truncate at apex, green 3-veined costa excurrent into a short awn ca. 0.6−0.8 mm long; pistillate glumes triangular-lanceolate, 5−7 mm long, membranous, glabrous, green 3-veined costa excurrent into a short awn for ca. 2 mm. Perigynia longer than the glumes, green, oval, obscurely trigonous, 6−8 × 3 mm, membranous, distinctly veined, pubescent on veins, gradually contracted into a ca. 2 mm long beak; orifice 2-lobed with long teeth. Nutlets tightly enveloped, rhombic-ovoid, trigonous, 4−5 mm long, brownish-black, with angles constricted at the middle, base shortly stipitate, apex abruptly contracted into an erect short beak 1 mm long; persistent style, base thickened. Flowering and fruiting January to May.

### Distribution and Habitat

*Carex jianfengensis* was collected from Jianfeng Ling Nature Reserve, Hainan, China. It grows under the tropical mountain rain forest at altitudes of 700–900 m. Associates include *Polyspora hainanensis* (H. T. Chang) C.X. Ye ex B.M. Barthol. & T.L. Ming, *Schima superba* Gardner & Champ., *Alsophila spinulosa* (Wall. ex Hook.) R.M. Tryon, *Schizostachyum pseudolima* McClure, *Hypolytrum nemorum* (Vahl) Spreng., *Carex breviscapa* C.B. Clarke, and *Dianella ensifolia* (L.) DC.

### Phenology

Flowering occurs from January and usually seeds mature in March to May.

### Etymology

The epithet “*jianfengensis*” refers to the type locality, Jianfeng Ling Natural Reserve.

### Conservation status

So far, this species is known from only one population and comprises approximately 2800 caespitose individuals, covers an area of 1000 m^2^. According to the IUCN (2001) category and criteria, *Carex jianfengensis* is a vulnerable species (VU).

### Relationships

The new species belongs to *Carex* sect. *Rhomboidales*. In Hainan Island, four species, *C*. *harlandii* Boott, *C*. *saxicola* Tang & F.T. Wang, *C*. *longipetiolata* Q.L. Wang, H.B. Yang & Y.F. Deng and *C*. *procumbens* H.B. Yang, X.X. Li & G.D. Liu have been reported[14,16]. In addition to the above, 5 new taxa from other regions have recently been described in sect. *Rhomboidales*: *C*. *jubozanensis* J. Oda & A. Tanaka, *C*. *austrozhejiangensis* C.Z. Zheng & X.F. Jin, *C*. *kagoshimensis* Tak. Shimizu, *C*. *yandangshanica* C.Z. Zheng & X.F. Jin and *C*. *paracheniana* X.F. Jin, D.A. Simpson & C.Z. Zheng[9,11–13,26]. However, *C*. *jianfengensis* can be easily distinguished from species mentioned above by its short culms less than 5 cm and its subbasal and approximate spikes. It is similar to *C*. *zunyiensis* based on the short culms, but differs have been mentioned in above Table 1.

#### Additional specimens examined (Paratypes)

CHINA. Hainan: Ledong County, Jianfeng Ling Nature Reserve, under forest, alt. 700−900 m, 15 April 2014, Yang Hubiao 20140415001 (two duplicates, TCGRI).
